# Microwave ablation therapy assisted by artificial pneumothorax and artificial hydrothorax for lung cancer adjacent to the vital organs

**DOI:** 10.3389/fonc.2022.981789

**Published:** 2022-08-23

**Authors:** Jian Chen, Liqin Qi, Jin Chen, Qingfeng Lin, Yuan Yan, Jie Chen, Zhengyu Lin

**Affiliations:** ^1^ Department of Interventional Radiology, First Affiliated Hospital of Fujian Medical University, Fuzhou, China; ^2^ Department of Endocrinology, Fujian Institute of Endocrinology, Fujian Medical University Union Hospital, Fuzhou, China; ^3^ Department of Interventional Radiology, Sanming Second Hospital, Sanming, China; ^4^ Fujian Provincial Key Laboratory of Precision Medicine for Cancer, First Affiliated Hospital of Fujian Medical University, Fuzhou, China

**Keywords:** lung cancer, artificial pneumothorax, artificial hydrothorax, microwave ablation, x-ray computed tomography

## Abstract

**Objectives:**

This study aimed to investigate the technical methods and safety of artificial pneumothorax and artificial hydrothorax in the treatment of lung cancer adjacent to vital organs by CT-guided microwave ablation.

**Subjects and Methods:**

Three of the six patients were men and three were women, with a mean age of 66.0 years (range 47–78 years). There patients had primary pulmonary adenocarcinoma, one had lung metastasis from liver cancer, one had lung metastasis from colon cancer, and one had lung metastasis from bladder cancer. There were four patients with a single lesion, one with two lesions, and one with three lesions. The nine lesions had a mean diameter of 1.1 cm (range 0.4–1.9). In three patients, the lung cancer was adjacent to the heart, and in the remaining three, it was close to the superior mediastinum. Six patients were diagnosed with lung cancers or lung metastases and received radical treatment with microwave ablation (MWA) assisted by artificial pneumothorax and artificial hydrothorax in our hospital. Postoperative complications were observed and recorded; follow-up was followed to evaluate the therapeutic effect.

**Results:**

The artificial pneumothorax and artificial hydrothorax were successfully created in all six patients. A suitable path for ablation needle insertion was also successfully established, and microwave ablation therapy was carried out. 2 patients developed pneumothorax after operation; no serious complications such as operation-related death, hemothorax, air embolism and infection occurred.Moreover, 4–6 weeks later, an enhanced CT re-examination revealed no local recurrence or metastasis, and the rate of complete ablation was 100%.

**Conclusions:**

Microwave ablation, assisted by artificial pneumothorax, artificial hydrothorax, is a safe and effective minimally invasive method for treating lung cancer adjacent to the vital organs, and optimizing the path of the ablation needle and broadening the indications of the ablation therapy

## Introduction

Lung cancer was the most common cancer in men, accounting for about 24.6% (549,800) of all new cancers in China;and was the second common cancer in women. And lung cancer was the most common cause of cancer death both for both sexes in China (1).Surgical resection remains the reference standard for the treatment of localized non-small cell lung cancer. However, only 20% of all diagnosed lung cancers are surgically resectable (2). Microwave ablation (MWA) has been proved to be safe and effective in the treatment of primary and metastatic lung tumor (3–5). MWA can be an alternative treatment

modality for early-stage NSCLC patients who are ineligible for surgery (6–10). When the lung lesions were adjacent to the vital organs (including the esophagus, trachea, heart, great vessels, diaphragm, and mediastinum), it was difficult to clearly show the effective puncture path and perform the ablation using a conventional CT scan. Alternatively, due to an insufficient safety margin, conventional percutaneous ablation is likely to cause thermal damage to the vital organs, resulting in serious complications. To address this technical issue, microwave ablation therapy with artificial pneumothorax, artificial hydrothorax, and body position adjustment was used for lung cancer adjacent to the vital organs, with satisfactory effects. The report is as follows.

## Materials and methods

### Clinical data

From November 2020 to March 2022, six patients were diagnosed with lung cancer adjacent to the vital organs (including the esophagus, trachea, heart, great vessels, diaphragm, and mediastinum) and received radical treatment with microwave ablation (MWA) assisted by artificial pneumothorax and artificial hydrothorax in our hospital. Three of the six patients were men and three were women, with a mean age of 66.0 years (range 47–78 years). There patients had primary pulmonary adenocarcinoma, one had lung metastasis from liver cancer, one had lung metastasis from colon cancer, and one had lung metastasis from bladder cancer. There were four patients with a single lesion, one with two lesions, and one with three lesions. The nine lesions had a mean diameter of 1.1 cm (range 0.4–1.9). In three patients, the lung cancer was adjacent to the heart, and in the remaining three, it was close to the superior mediastinum. Inclusion criteria were as follows: ① patients with primary or metastatic lung cancer confirmed by puncture histological biopsy or imaging examination; ② patients who could not tolerate surgical resection due to poor cardiopulmonary function or old age; ③ patients who refused surgical resection; ④ patients who had a new or residual lesion after surgical resection and were unable to tolerate or refused re-surgery; ⑤ patients with multiple ground-glass nodules (GGNs) (ablation of the primary lesion first before considering the ablation of the other lesions according to the progression); ⑥ patients with lesions adjacent to the vital organs, with a distance ≤ 0.5 cm, and were more likely to suffer thermal damage to the vital organs; and ⑦ patients with a total number of unilateral lung metastases ≤3, total number of bilateral lung metastases ≤5, and maximum tumor diameter ≤3 cm. Exclusion criteria were as follows: ① patients suffering from severe cardiac, hepatic, pulmonary, or renal failure, ② patients with platelet count <50×10^9^/L, ③ patients with severe bleeding proclivity and coagulation dysfunction that could not be corrected in a timely manner, and ④ patients with severe pulmonary fibrosis and pulmonary hypertension. **Table 1** shows the general conditions of the patients.

**Table 1:** Clinical data of 6 patients with lung cancer and the results of microwave ablation therapy assisted by artificial pneumothorax and artificial hydrothorax.

Number	Gender	Age (year)	Primary tumor and pathological classification	tumor stage	Number of tumors (pcs)	maximum tumor diameter (cm)	Artificial pneumothorax volume (ml	Artificial pleural effusion volume (ml)	complication
1	woman	72	pulmonary adenocarcinoma	I	2	1.7	200	400	none
2	man	55	colon cancer	IV	1	1.0	200	200	pneumothorax
3	woman	68	bladder cancer	IV	1	1.3	200	400	none
4	woman	76	pulmonary adenocarcinoma	I	1	1.9	200	300	none
5	man	47	liver cancer	IIIb	3	1.0	300	500	pneumothorax
6	man	78	pulmonary adenocarcinoma	I	1	1.5	100	500	none

### Instruments and equipment

①64 fit multi-slice spiral computed tomography (CT) scanner (Siemens SOMATOM definition AS, Germany). ②MTC-3C MWA system (Vision-China Medical Devices R&D Center/KY-2450B MWA system (Nanjing Great Wall Institute for Application of Microwave Energy), the microwave emission frequency is (2450 ± 50) MHz, and the output power is 0–100 W. ③Water-cooled MWA antenna (effective length is 100–180 mm, outer diameter is 15–18 G, and emission length of the end microwave is 15 mm). ④Catheter (8F-20cm, Guangdong Baihe Medical Technology Co.,Ltd)

### Preoperative preparation

**Figure 1:**
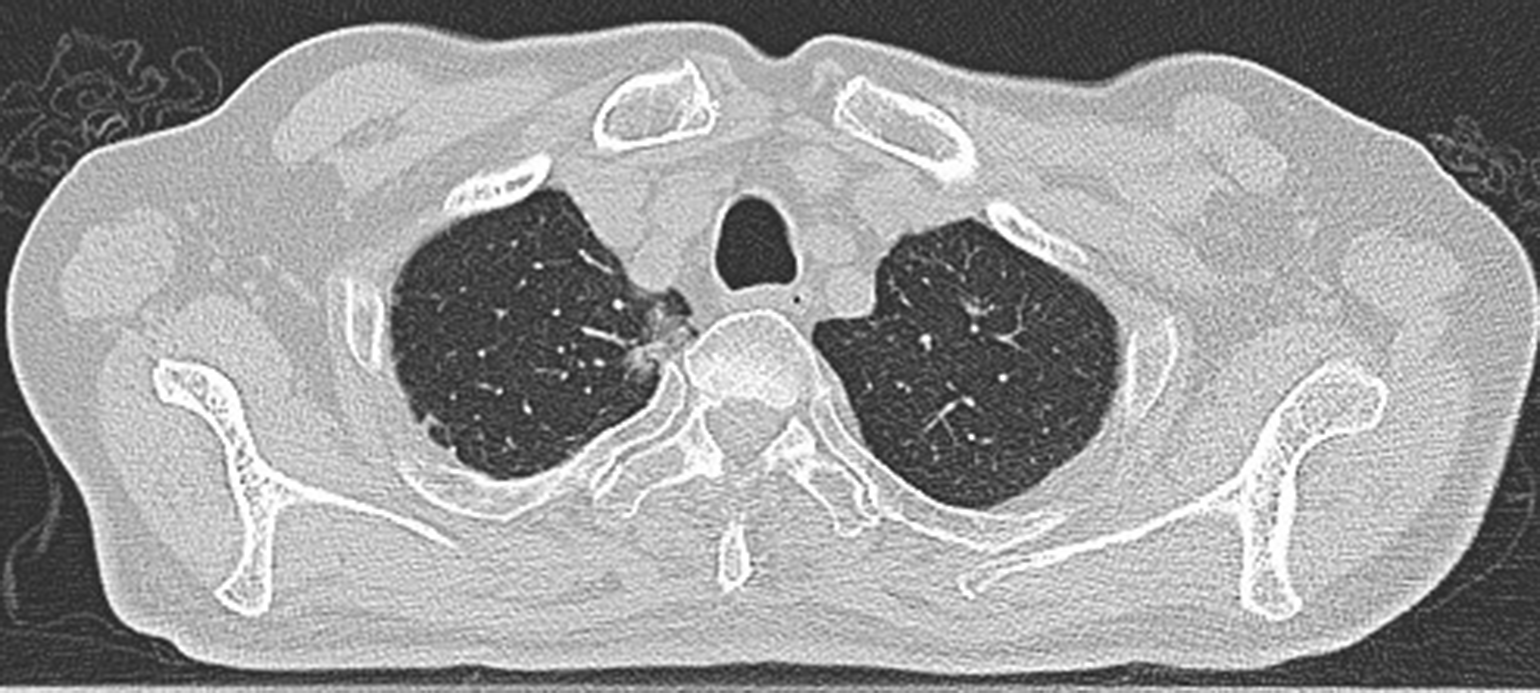
Preoperative scan: Preoperative CT showed a nodule in the right upper lobe. Invasive adenocarcinoma of the right upper lobe of a 78-year-old man.

The size, location, and adjacent important organs and blood vessels of the lesions were detected by conventional CT plain scans and enhanced examinations within 2 weeks before the operation (**Figure 1**). Blood routine examinations and coagulation time were evaluated to exclude hemorrhagic diseases. The patients fasted for 4 h before the operation, and the venous channels were opened.The patients underwent breath−holding training according to the preoperative imaging data.

### Operating procedures

#### Artificial pneumothorax

**Figure 2:**
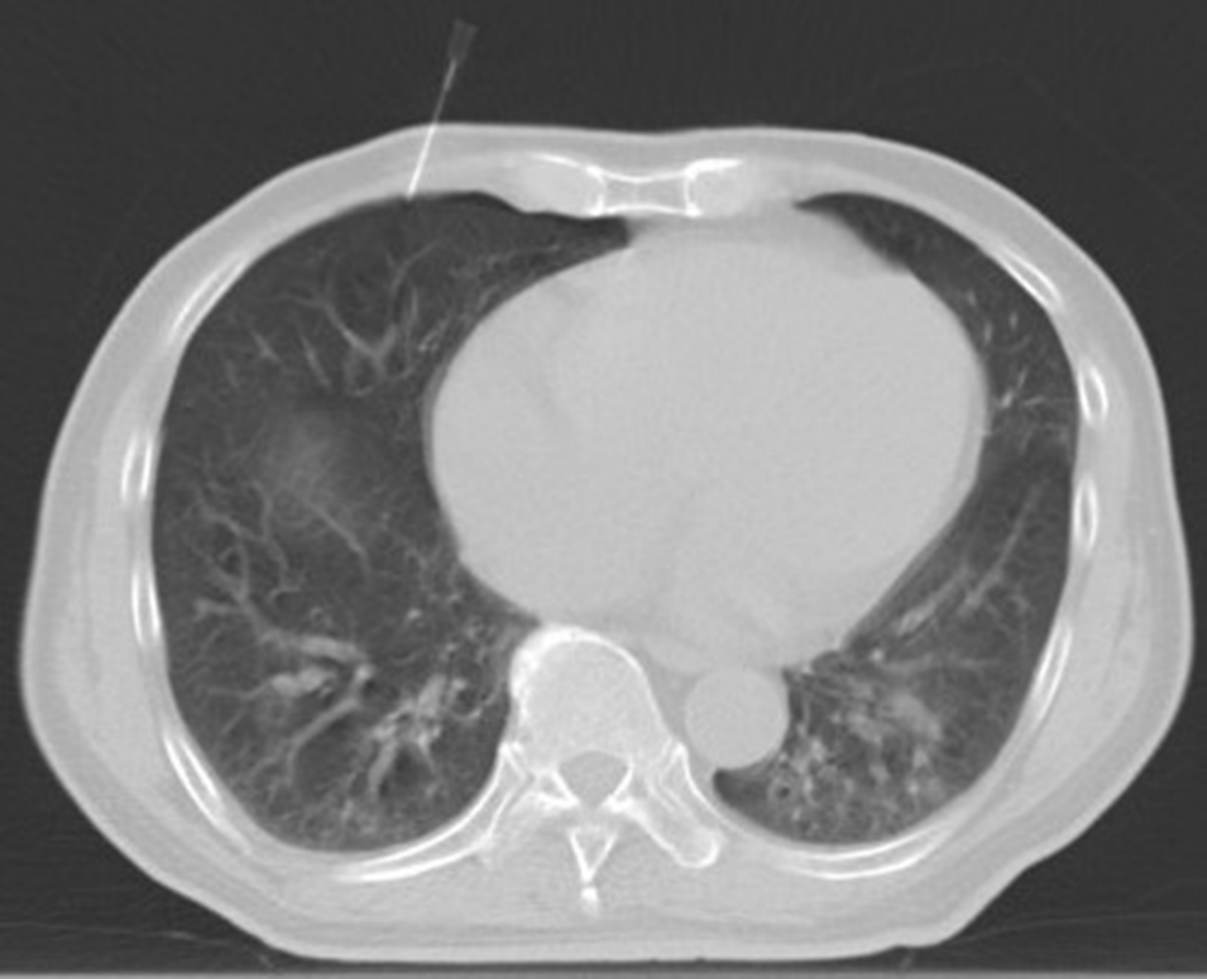
The artificial pneumothorax was created: Intraoperative scan was acquired with the patient in the supine position. the examination couch was adjusted to the level of the diaphragm dome on the puncture side. When the bevel tip of the 5-ml syringe needle reached the pleura, the syringe would be removed, a transparent rubber tube would be connected, and a little sterile saline would be injected into the tube to form a water column.

**Figure 3:**
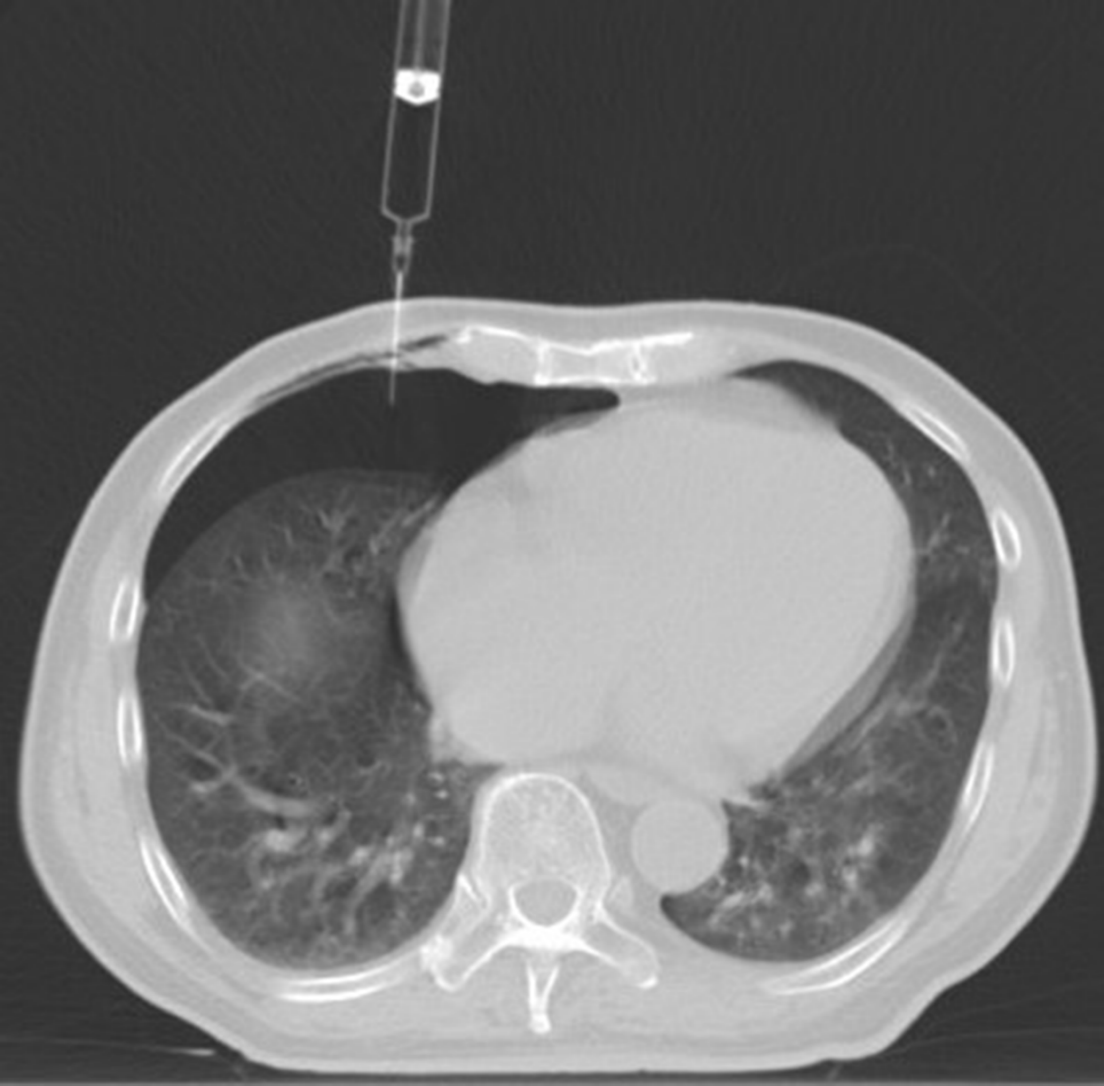
When the needle tip broke through the parietal pleura and the water column was drawn into the pleural cavity containing certain negative pressure, and an appropriate amount of air (100 ml) would be injected into the pleural cavity.

The patient was positioned supine, the examination couch was adjusted to the level of the diaphragm dome on the puncture side, and a positioning fence was taped to the body surface where the needle was to be inserted. The laser positioning light was activated, and the needle insertion site was determined while keeping the heart in mind. Before the puncture, routine disinfection and placement of surgical drape were performed, as well as layer-by-layer infiltration anesthesia with 2% lidocaine. When the bevel tip of the 5-ml syringe needle reached the pleura, the syringe would be removed, a transparent rubber tube would be connected, and a little sterile saline would be injected into the tube to form a water column (**Figure 2**); when the needle tip broke through the parietal pleura and the water column was drawn into the pleural cavity containing certain negative pressure, the rubber tube would be removed, a three-way tube and a 50-ml syringe would be connected, and an appropriate amount of air (100–300 ml) would be injected into the pleural cavity (**Figure 3**). Following that, the patient was evaluated for chest tightness, shortness of breath, and other uncomfortable symptoms. The three-way tube was closed after the artificial pneumothorax was created.

#### Artificial hydrothorax

**Figure 4:**
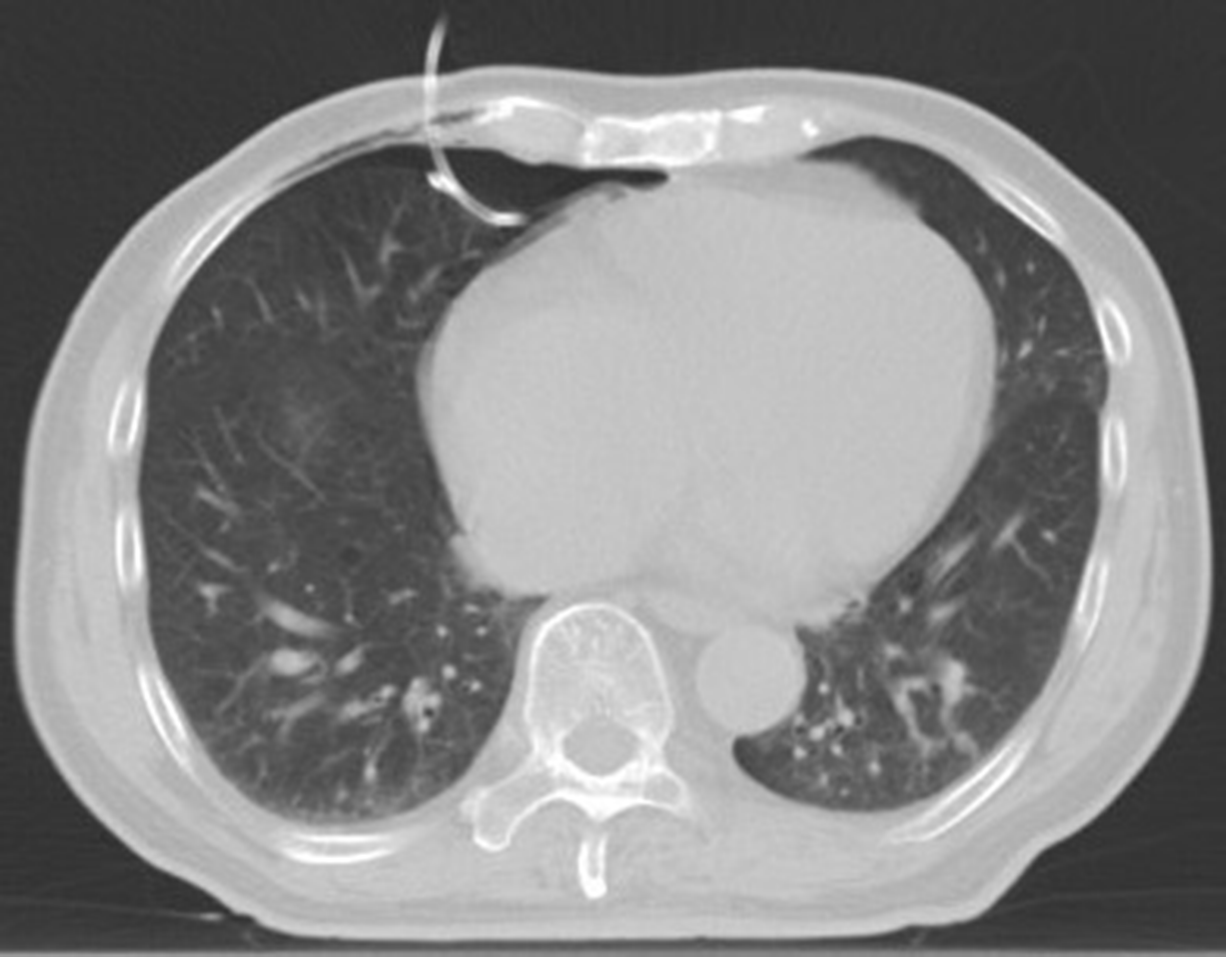
The artificial hydrothorax was created: Following the establishment of the artificial pneumothorax, the guide wire was inserted after the puncture needle gradually reached the pleural cavity containing the artificial pneumothorax, and an 8F central venous catheter was indwelled along the guide wire.

**Figure 5:**
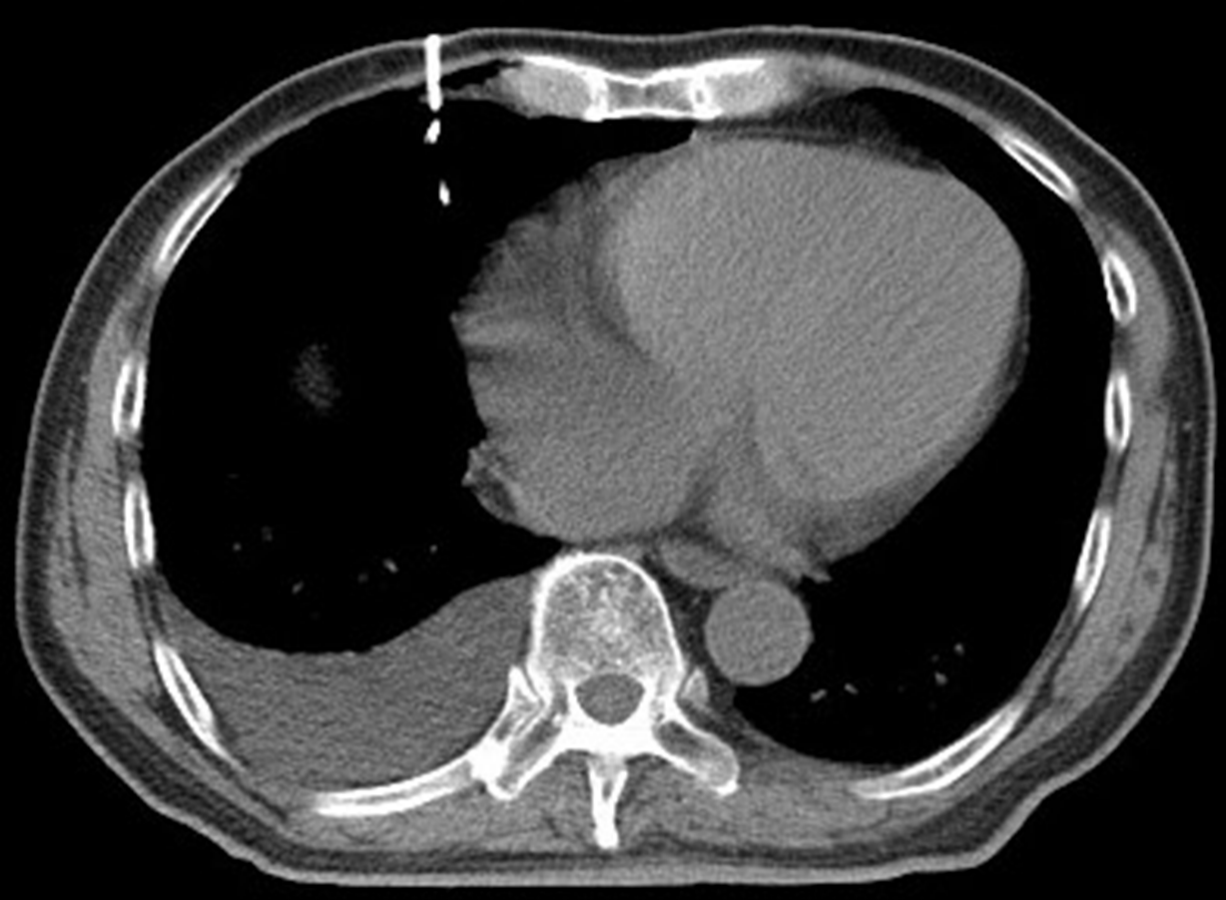
To isolate the lesion and the adjacent vital organs, an appropriate amount of normal saline (500 ml) was injected into the catheter to form an isolation belt.

Following the establishment of the artificial pneumothorax, a positioning fence is attached to the proposed tube placement site on the body surface, and the laser positioning light is activated to determine the needle entry point. Before the puncture, routine disinfection and placement of surgical drape were performed, as well as layer-by-layer infiltration anesthesia with 2% lidocaine. The guide wire was inserted after the puncture needle gradually reached the pleural cavity containing the artificial pneumothorax, and an 8F central venous catheter was indwelled along the guide wire (**Figure 4**). To isolate the lesion and the adjacent vital organs, an appropriate amount of normal saline (200–500 ml) was injected into the catheter to form an isolation belt (**Figure 5**). The injection process should be done slowly in order to see if the patient develops chest tightness, shortness of breath and other symptoms. It was decided to make an artificial hydrothorax.

### Microwave ablation therapy

**Figure 6:**
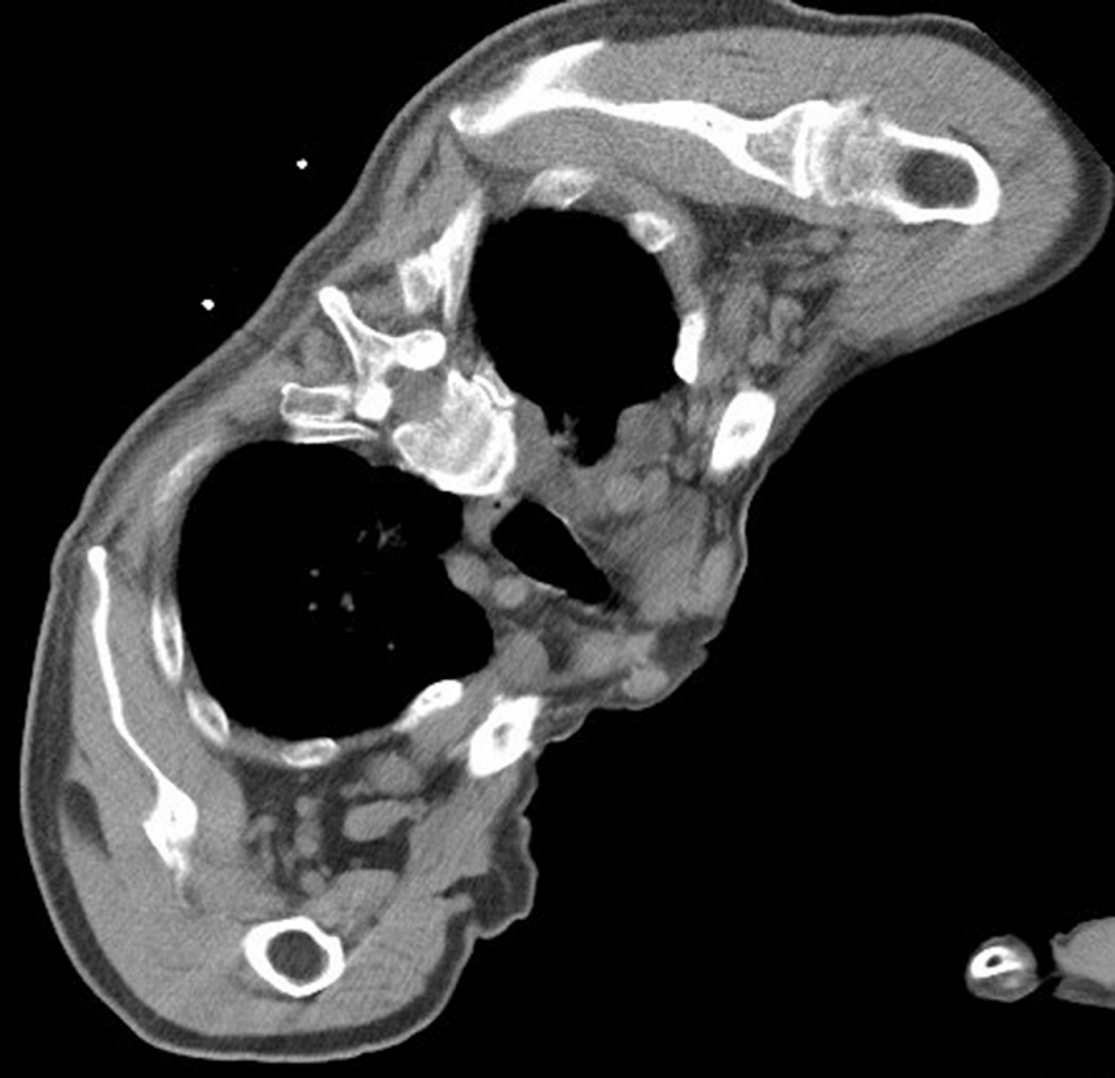
Intraoperative scan: the patient’s body position was adjusted to isolate the lesion and the adjacent vital organs, and the appropriate amount of normal saline was to form an isolation belt.

**Figure 7:**
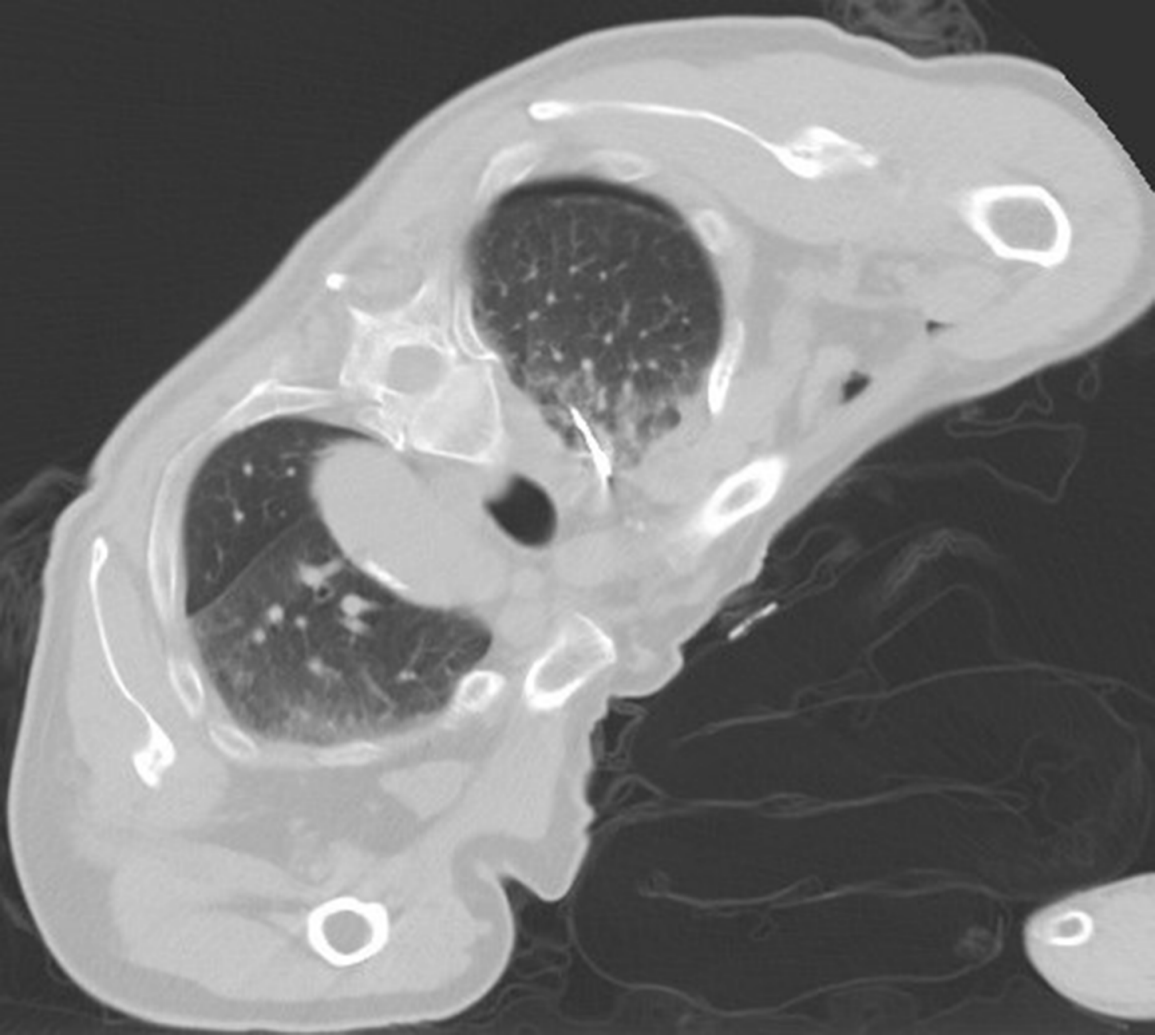
Guided by CT, the needle was gradually inserted through the microwave antenna to the bottom of the lesion for ablation.

**Figure 8:**
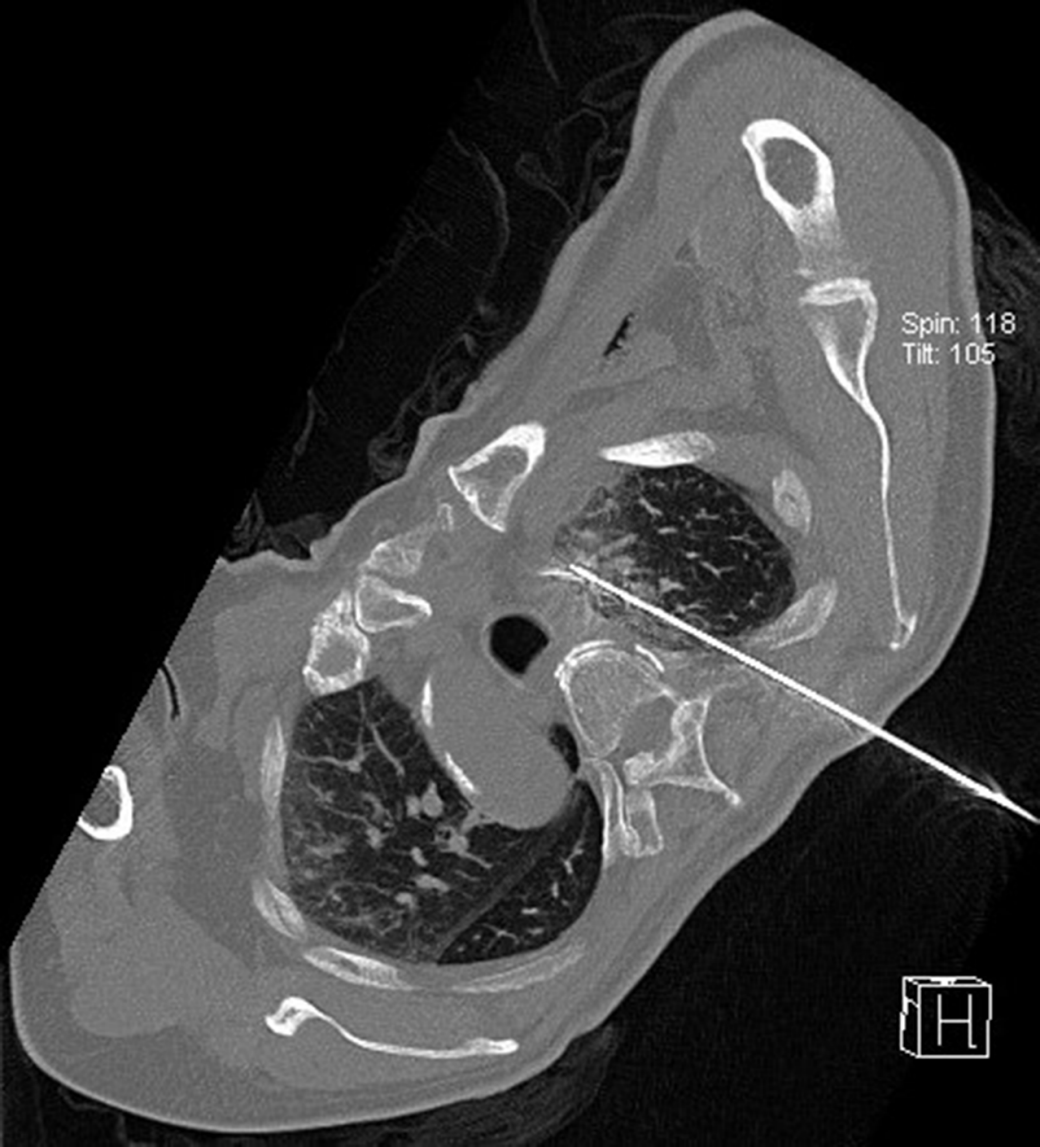
Intraoperative scan: the Reconstructed CT image.

**Figure 9:**
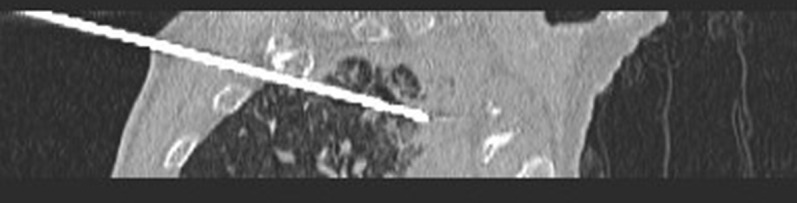
Intraoperative scan: the Reconstructed CT image.

**Figure 10:**
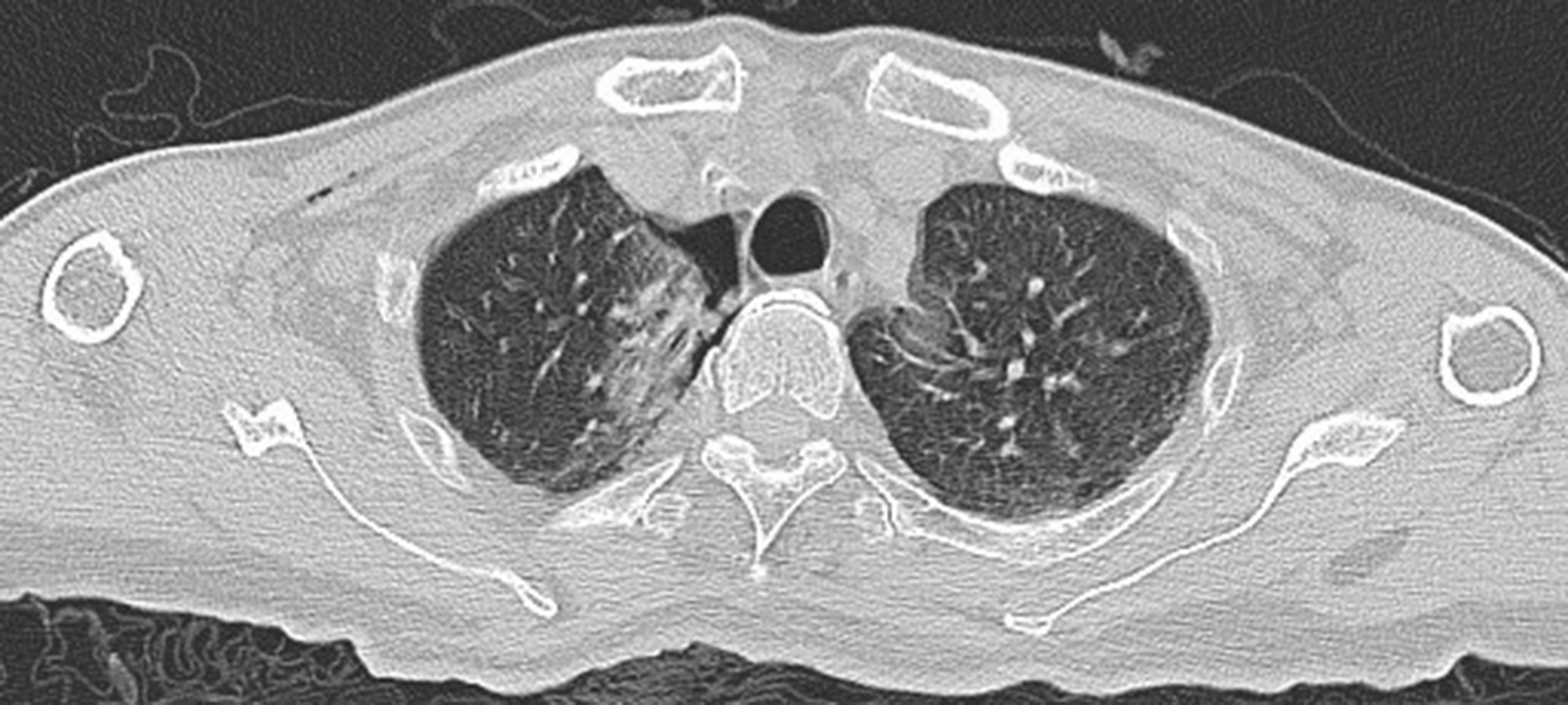
Post-ablation scan: The lesion was achieved complete ablationthe: post-ablation ground-glass opacity around the post-ablation target zone should be at least 5 mm greater than the boundary of the gross tumor region.

Following the establishment of the artificial pneumothorax and artificial hydrothorax, the size, location, blood flow, and other characteristics of the tumor were reconfirmed using the preoperative CT of the patient, and the patient’s body position was adjusted to the optimal puncture position (**Figure 6**). Next, guided by CT, the needle was gradually inserted through the microwave antenna to the bottom of the lesion for ablation (**Figures 7**–**9**). The appropriate microwave output power and ablation time were chosen based on the tumor size and shape. The preset microwave ablation power was set to 50–80 W, and the ablation time was limited to 20 min (**Figure 10**).

### Immediate efficacy evaluation and follow-up

Evaluation of immediate efficacy (11) was as follows: ① preliminary evaluation of the success of the operating technique and ② observation of the ablation boundary. Recommendation: For complete ablation, the post-ablation ground-glass opacity around the post-ablation target zone should be at least 5 mm greater than the boundary of the gross tumor region (GTR); ③ Complications should be observed.

**Figure 11:**
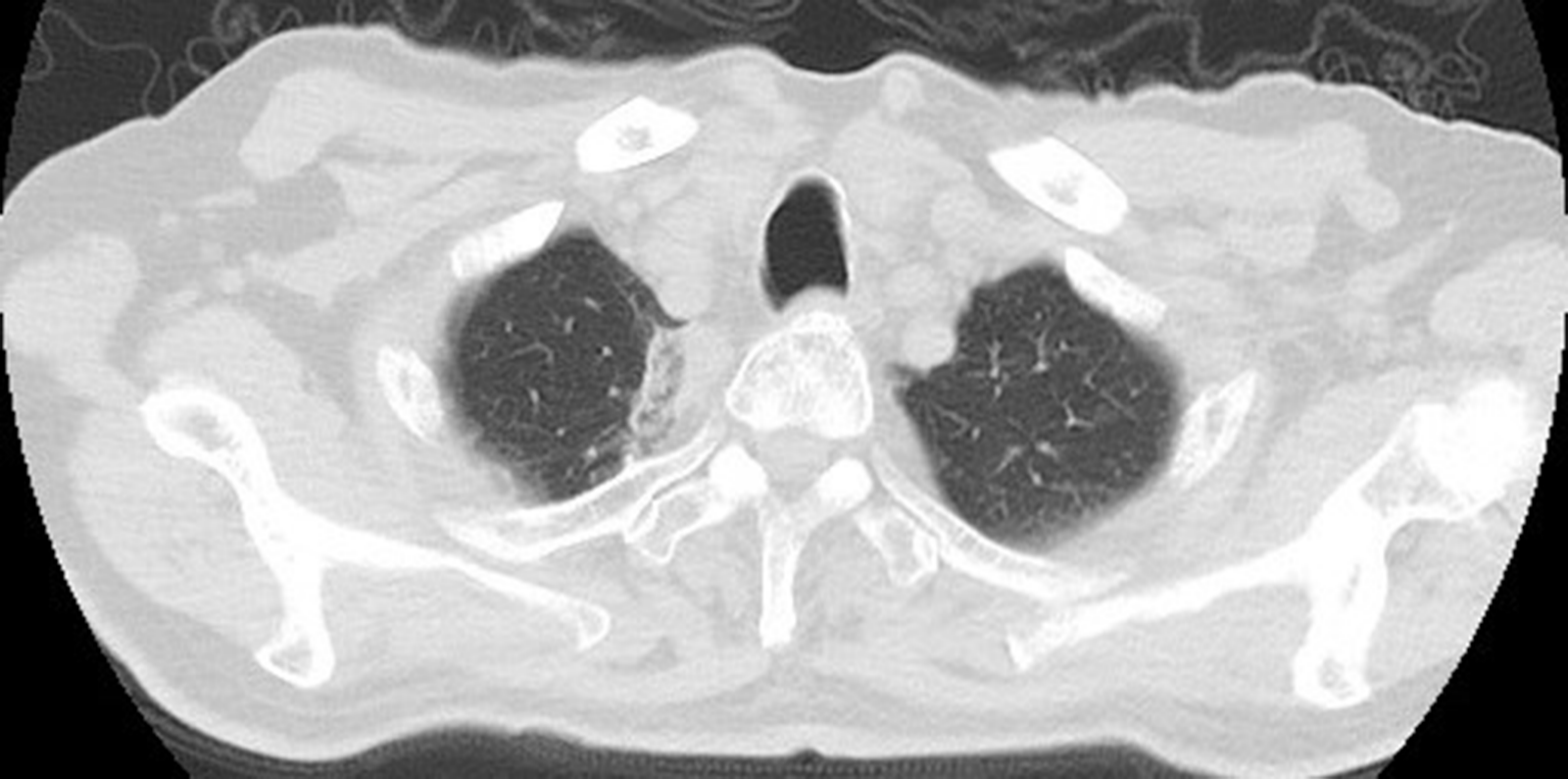
Cross-sectional image:Follow-up after 1 month found that the lesions were completely ablated, and no tumor lesions remained or recurred.

**Figure 12:**
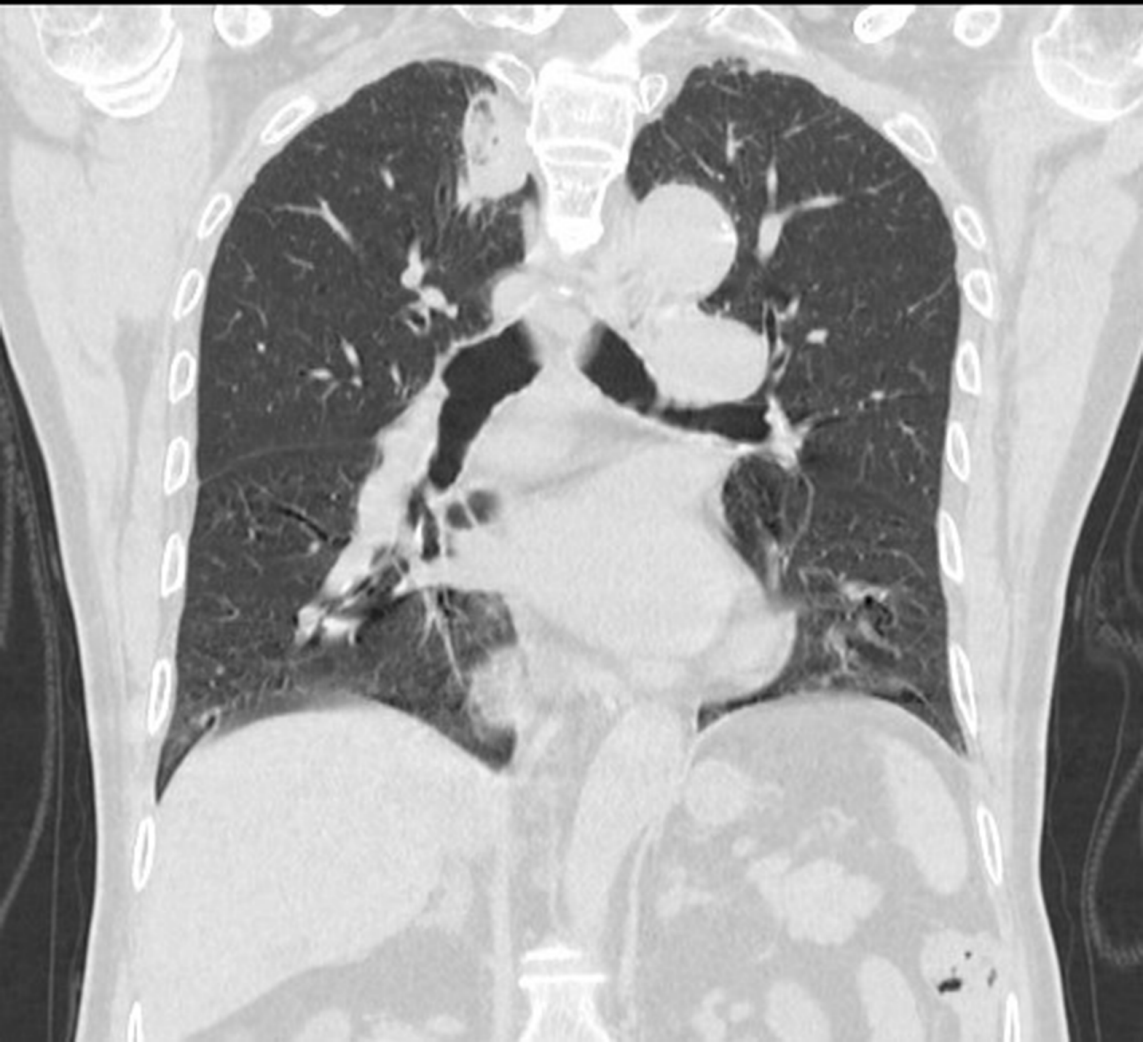
Coronal image:Follow-up after 1 month found that the lesions were completely ablated, and no tumor lesions remained or recurred.

Local therapeutic efficacy evaluation (12, 13): The lesion on the enhanced chest CT re-examination 4–6 weeks after surgery served as the baseline for judging efficacy. ① Complete ablation (presence of any of the following manifestations): disappearance of the lesion, complete formation of the cavity, fibrosis of the lesion (scar), reduction, no change or enlargement of the solid nodule (no signs of abnormal enhancement of the contrast agent on the CT scan), atelectasis (absence of any signs of abnormal enhancement of the contrast agent on the CT scan of the lesion in the atelectasis) (**Figures 11**, **12**). ② Incomplete ablation (presence of any of the following manifestations): (a) On the cavity formation and fibrosis edges of the lesion, there were still typical imaging manifestations of GGN; (b) there was fibrosis in part of the lesion, but there were still some solid components, and the solid part showed enhancement on the CT scan and/or the tumor showed metabolic activity on the positron emission tomography-computed tomography (PET-CT) scan; (c) the solid nodule did not change in size or enlargement, but it did show signs of abnormal contrast enhancement on CT scan and/or abnormal metabolic activity on PET-CT scan.

### Statistical methods

Data were analyzed using IBM SPSS Statistics for Windows, version 22.0 (IBM Corp., New York). Measurement data were expressed as means ± standard deviation, whereas counting data were expressed as percentages and were compared by Chi-square tests. P <0.05 was considered a statistically significant difference.

## Results

### Ablation results

After injections with a mean amount of gas of 200.0 ml (range 100–300) and a mean amount of normal saline of 383.3 ml (range 200–500), the artificial pneumothorax and artificial hydrothorax were successfully created in all six patients. A suitable path for ablation needle insertion was also successfully established, and microwave ablation therapy was carried out. For the creation of the artificial pneumothorax and artificial hydrothorax, the surgery time increased by an average of 21.5min (range 16.5–39.6), and the number of local scans increased by an average of 8 times (range 5–12). The conventional CT scan performed 3 days after ablation confirmed that all lesions had been completely abated. Moreover, 4–6 weeks later, an enhanced CT re-examination revealed no local recurrence or metastasis, and the rate of complete ablation was 100%.

### Complication

All patients tolerated the surgery. two patients developed pneumothorax after operation, which improved after intubation and drainage; no serious complications such as operation-related death, hemothorax, air embolism and infection occurred.

## Discussion

As a precise and minimally invasive technique, local microwave ablation has grown in popularity in recent years. It has been used to treat lung cancer, especially early-stage lung cancer, with remarkable therapeutic results (3–10, 14). MWA operates under the action of a microwave electromagnetic field: the polar molecules in the tumor tissue, such as water molecules and protein molecules, vibrate at an extremely high speed, causing the collision and friction between molecules, producing a high temperature of up to 60°C–150°C in a short period of time, and resulting in coagulative necrosis of cells. Concentrated in a certain range by the radiator, the microwave energy can be effectively radiated to the desired target zone. Thermal microwave radiation causes more convection and less thermal precipitation in the lung (12, 13, 15). Therefore, this technique has a minimal invasion, definite curative effect, high safety, strong repeatability, and suitability for a wide range of people.

The artificial pneumothorax or artificial hydrothorax is a useful adjunct in the clinical treatment of various diseases. Professor Lin Zhengyu and his team successfully performed puncture biopsy in 11 patients with mediastinal lesions, using artificial pneumothorax and body position adjustment, with no pneumothorax or bleeding complications (16). Moreover, artificial pneumothorax is a safe and effective method for pain relief during MWA of subpleural lung tumors (17, 18). In addition, artificial pneumothorax or hydrothorax has been used with significant success in thermal ablation of tumors adjacent to lung tissue in the lower lobe or near the diaphragm dome (19–21). In this study, because the lung lesions were adjacent to the vital organs, it was difficult to clearly show the effective puncture path and perform the ablation using a conventional CT scan. Alternatively, due to an insufficient safety margin, conventional percutaneous ablation is likely to cause thermal damage to the vital organs, resulting in serious complications. The introduction of artificial pneumothorax and artificial hydrothorax not only solves this problem but also broadens the scope of microwave ablation therapy. It is easy to create an artificial pneumothorax, and it is also simple to separate lesions from adjacent structures using an artificial pneumothorax and body position adjustment. However, after the artificial pneumothorax was established, the isolated local visceral pleura becomes significantly more elastic, and the local pulmonary motion increases, making the pulmonary nodules more likely to displace during the puncture with ablation needle. And because such displacement makes puncturing the pulmonary nodules more difficult, it is often difficult to puncture into the center of the lesion for ablation. When compared to artificial pneumothorax, isolation with artificial hydrothorax has less impact on the elasticity of the local visceral pleura and local pulmonary motion, making it easier to puncture the pulmonary nodules, allowing for puncture into the center of the lesion for ablation and improving the efficacy of ablation. Because creating artificial hydrothorax under CT guidance is relatively difficult, artificial pneumothorax is often created before artificial hydrothorax; if complicated with pleural hemorrhage, artificial hydrothorax may affect judgment of hemothorax. Therefore, artificial pneumothorax was used in combination with artificial hydrothorax in this study to successfully assist in the microwave ablation of lung cancer in six patients. This combination therapy not only provided a good puncture path for ablation, but it also protected the adjacent vital organs from thermal damage during thermal ablation, resulting in remarkable results. Excessive gas or liquid in the pleural cavity will impair respiratory functions and may cause chest tightness, shortness of breath, dyspnea, and chest pain, especially in patients with poor lung function. In this study, the amount of injected gas and liquid was reduced by adjusting the body position of patients. There was no death or other special disease associated with surgery following ablation therapy, and there was no serious cardiopulmonary disease caused by the large amount of gas and liquid injected. These findings are sufficient to demonstrate the high safety of ablation therapy, which is assisted by artificial pneumothorax, artificial hydrothorax, and body position adjustment.

In this study, the rate of complete ablation was 100%, only two patients developed pneumothorax, and there were no serious complications such as ablation-related bleeding or infection after surgery, confirming that artificial pneumothorax combined with artificial hydrothorax and body position adjustment is extremely safe and effective. Artificial pneumothorax combined with MWA has the limitation that patients with pleural adhesions occurring after pulmonary surgery or lung cancer radiotherapy are not suitable for this therapy (18).Pleural adhesions can prevent sufficient lung tissue from being compressed and can also cause pleural tearing and bleeding. However, because the preoperative plain scan cannot always detect pleural adhesions, the degree and location of adhesions can only be determined after a certain amount of gas is injected and the lung tissue retracts.

## Limitations

First, this is a single-center retrospective study with a small sample size and no statistical significance; the long-term efficacy and corresponding influencing factors should be investigated further in a controlled study with a large sample size. Second, in this study, the use of artificial pneumothorax and artificial hydrothorax increased surgery time and the number of local scans, which was time−consuming and increased the ionizing radiation received by patients. In addition, the follow−up of some patients was not regular

## Conclusion

To summarize, microwave ablation, assisted by artificial pneumothorax, artificial hydrothorax, and body position adjustment, is a safe and effective minimally invasive method for treating lung cancer adjacent to the vital organs.

## Data availability statement

The original contributions presented in the study are included in the article/supplementary materials. Further inquiries can be directed to the corresponding author.

## Ethics statement

The studies involving human participants were reviewed and approved by the ethics committee of First Affiliated Hospital of Fujian Medical University. The patients/participants provided their written informed consent to participate in this study.

## Author contributions

ZL conceptualized the study, prepared figures and tables. JianC wrote the article and prepared figures and tables. LQ collected the data, carried out the analysis, and prepared the figures and tables. JinC and QL participated in drafting and editing the article and assisted in the preparation of figures and tables. YY and JieC participated in figure preparation and drafting and editing the article. All authors contributed to the article and approved the submitted version.

## Funding

This work was supported by the Start-up Fund for Scientific Research, Fujian Medical University (Grant number: 2017XQ1094).

## Conflict of interest

The authors declare that the research was conducted in the absence of any commercial or financial relationships that could be construed as a potential conflict of interest.

## Publisher’s note

All claims expressed in this article are solely those of the authors and do not necessarily represent those of their affiliated organizations, or those of the publisher, the editors and the reviewers. Any product that may be evaluated in this article, or claim that may be made by its manufacturer, is not guaranteed or endorsed by the publisher.
